# Typology of how ‘harmful commodity industries’ interact with local governments in England: a critical interpretive synthesis

**DOI:** 10.1136/bmjgh-2022-010216

**Published:** 2023-01-23

**Authors:** Sarah McKevitt, Martin White, Mark Petticrew, Carolyn Summerbell, Milica Vasiljevic, Emma Boyland, Steven Cummins, Anthony A Laverty, Cornelia Junghans, Christopher Millett, Frank De Vocht, Eva Hrobonova, Eszter P Vamos

**Affiliations:** 1 Public Health Policy Evaluation Unit, School of Public Health, Imperial College London, London, UK; 2 MRC Epidemiology Unit, University of Cambridge School of Clinical Medicine, Cambridge, UK; 3 PHP, London School of Hygiene and Tropical Medicine, London, UK; 4 Fuse - Centre for Translational Research in Public Health, Newcastle University, Newcastle upon Tyne, UK; 5 Department of Sport and Exercise Sciences, Durham University, Durham, UK; 6 Department of Psychology, Durham University, Durham, UK; 7 Department of Psychology, University of Liverpool, Liverpool, UK; 8 Population Health Innovation Lab, Department of Public Health, London School of Hygiene & Tropical Medicine, London, UK; 9 Population Health Sciences, University of Bristol, Bristol, UK; 10 NIHR Applied Research Collaboration West, Bristol, UK; 11 Westminster City Council, London, UK

**Keywords:** Public Health, Review, Health policies and all other topics, Health policy, Prevention strategies

## Abstract

**Introduction:**

Industries that produce and market potentially harmful commodities or services (eg, tobacco, alcohol, gambling, less healthy foods and beverages) are a major influence on the drivers of behavioural risk factors for non-communicable diseases. The nature and impact of interactions between public bodies and ‘harmful commodity industries’ (HCIs) has been widely recognised and discussed at national and international levels, but to date little is known about such interactions at local or regional government levels. This study aimed to identify and characterise actual and potential interactions and proposes a typology of interactions between HCIs and English local authorities (LAs).

**Methods:**

Five electronic databases covering international literature (PubMed, EBSCO, OVID, Scopus and Web of Science) were searched up to June 2021. We also performed online searches for publicly available, web-based grey literature and documented examples of interactions in an English LA context. We conducted a critical interpretive synthesis of the published and grey literature to integrate and conceptualise the data in the context of English LAs.

**Results:**

We included 47 published papers to provide the frame for the typology, which was refined and contextualised for English LAs through the available grey literature. Three categories were developed, describing the medium through which interactions occur: (1) direct involvement with LAs, (2) involvement through intermediaries and (3) involvement through the local knowledge space. Within these, we grouped interactions into 10 themes defining their nature and identified illustrative examples.

**Conclusion:**

Our typology identifies complex inter-relationships and characterises interactions between HCIs and LAs, with illustrative examples from English LAs. Drawn from well-established theories and frameworks in combination with contextual information on English LAs, this typology explores the LA perspective and could help local decision-makers to maximise population health while minimising negative impacts of HCIs.

**PROSPERO registration number:**

CRD42021257311

**WHAT IS ALREADY KNOWN ON THIS TOPIC**

- Increases in preventable non-communicable diseases are largely driven by the consumption and use of tobacco, alcohol, gambling and less healthy foods and drinks, produced by ‘harmful commodity industries’ (HCIs).
- Public bodies often have complex and close inter-relationships with HCIs, but the manifestations of these interactions have not been characterised at regional or local government level in England nor elsewhere.

**WHAT THIS STUDY ADDS**

- Our typology of interactions between HCIs and local government, intermediaries and the local knowledge space, acknowledges the perspective of local government in seeking interactions with the private sector for mutual benefit, which does not apply to interactions with HCIs.

**HOW THIS STUDY MIGHT AFFECT RESEARCH, PRACTICE OR POLICY**

- The typology identifies complex inter-relationships and could help inform commercial policies and further considerations for decision-makers in local government, their intermediaries and the local knowledge space on how to maximise population health and minimise negative impacts of HCI interactions.

## Introduction

‘Harmful commodity industries’ (HCIs) are corporations that produce and market products and services such as tobacco, alcohol, gambling and unhealthy foods and beverages. Use of such harmful commodities is a major driver of the burden of preventable non-communicable diseases (NCDs), and HCIs have been identified as important vectors of the growth of NCDs.^1–4^ Addressing the commercial determinants of health (CDoH) is therefore a pressing public health priority.^5^ The jobs, revenue, technical and research expertise, and ability to exert corporate power provide HCIs with a powerful voice to shape public perceptions of health, science and policy.^2^ The power of HCIs in influencing and shaping public health policy at the national and international level is well established^6–13^ through strategies to engage, and influence the cultural, social, political and knowledge environments.^6–8 13^ Approaches across HCIs are largely coherent,^14^ including echoing tactics of lobbying, marketing and corporate social responsibility (CSR) strategies, among others.^1 2 7 14 15^ These activities all extend and amplify HCI impact and influence, by enhancing the desirability and acceptability of harmful commodities, challenging policy barriers, deflecting attention from harms and whitewashing tarnished reputations of HCIs.^6^

A tension exists between HCI activities to encourage consumption of their products and maximise profits, and public health measures to reduce the burden of NCDs.^7 8 16 17^ The controversial nature of interactions between HCIs and public health is well recognised and discussed,^1 9 17–19^ including tobacco, alcohol and increasingly food industries^1 7–11^ in a range of contexts; globally and transnationally,^20 21^ in low-income and middle-income countries (LMICs),^22 23^ national organisations,^24^ academic environments,^25^ social media platforms^26^ and on explicit policies.^27 28^ In addition, recent attention also recognises the gambling industry, which is associated with significant harms including suicide, poor mental health and financial harms.^29–31^ Partnerships with gambling industry-funded bodies raise similar concerns to other HCIs.^32–34^

There are 333 English local authorities (LAs) split into two tier (county and district councils) and single tier (unitary authorities, metropolitan districts and London boroughs) structures to determine the council responsibilities. Article 5.3 of the WHO Framework Convention on Tobacco Control (FCTC)^35^ provides a key example of implementation of regulatory safeguards to protect public health policy from the interests of the tobacco industry at different levels of governance, including LAs. LAs have a legal obligation to act according to the FCTC, which sets a defined line for regulatory safeguards and enables local tobacco policy implementation while protecting LAs from legal challenges.^35^ As per the Health and Social Care Act 2012,’ LAs in England are responsible for all the public services and facilities in a particular area and obligated to improve the health of their population and promote healthy communities (Health and Social Care Act, 2012; national Planning Policy Framework, 2012). The devolution of public services shifting from central to local government, offers LAs the power to be better aligned with, and increase their sensitivity to specific local areas and local community needs.^36^ Close relationships between LA actors and the private sector are important for many reasons. These include generating revenue for LAs, boosting local investments and developments, providing resources and expertise, and sharing risks in developments, allowing LAs to deliver their key functions and vital services. As such, complex and sometimes close, inter-relationships between LAs and HCIs might not be perceived differently from other business relations, on the assumption that the interactions are equally mutually beneficial.

The complex and close inter-relationships between public bodies and HCIs are well established, but the manifestation of such activities at a regional or local government level, has not been explored. In addition, while previous research mainly focuses on the activities and the influence of HCIs, little is known about interactions between HCI and public bodies, in which LAs seek involvement from HCIs. Little is known about the nature and extent of interactions between LAs and HCIs. Therefore, it is critical to develop a comprehensive conceptualisation of interactions that considers and explores the LA perspective to inform decision-makers and ensure protection of populations from the harmful effects of HCIs. Understanding these interactions is particularly pertinent in the current economic climate as LAs operate under increasing financial constraints, and their investment, efficiency and retrenchment strategies may increasingly involve HCIs in attempts to compensate for reductions in public funding over the past decade.^37–39^ This study aimed to develop a typology that could encompass all potential interactions between HCIs and English LAs. We had two specific objectives: (1) synthesise the published international literature and develop a typology of the potential ways HCIs and LAs interact and (2) contextualise and refine the typology through the identification of documented examples of such interactions at the LA level in England.

## Methods

We adopted principles from critical interpretive synthesis (CIS)^40^ to guide the study methods. CIS is an approach explicitly oriented towards theory building through a dialectic process combining evidence and theory. Adopting a comprehensive critical narrative allowed us to integrate and interpret a diverse body of evidence into a coherent conceptual framework^40^ in a dynamic and iterative process. The protocol was registered with PROSPERO (CRD42021257311).

### Public involvement

Five members of the public were involved throughout the research process, including methodological planning and contributing to data collection methods. Crucially, they contributed to the appropriateness of the study framing and language, essential for engaging non-public health practitioners and enhancing the practical usability of the research.

### Reviewing the published literature

Although this study was not specifically a systematic or scoping review, we adopted a flexible systematic approach to our knowledge synthesis. We tailored and adapted the Preferred Reporting Items for Systematic Reviews and Meta-Analyses Extension for Scoping Reviews (online supplemental table 1) to guide the methodology.^41^

10.1136/bmjgh-2022-010216.supp1 Supplementary data

### Search strategy

The search strategy was guided using previously identified key papers which draw on well-documented HCI activities.^1 2 6 11 13 25 42–45^ The search strategy was developed for use in PubMed, using existing reviews, which explored for example, conflicts of interest in public health,^43^ the commercial determinants of obesity^44^ and corporate-political activity (CPA) of the food industry,^46^ and combined these with common terminology, Medline Subject Headings (MeSH) terms and free text, including synonyms, substitutes and plurals, to increase the search specificity and sensitivity. The search strategy was pilot tested using previously identified key papers, then independently tested by a second reviewer (EPV) and adapted appropriately for each additional database (online supplemental table 2).

Articles had to fulfil the predefined study eligibility criteria with the inclusion of papers published in a peer-reviewed journal, written in English, referring to humans and designed to collate (review, frame, typify, classify, conceptualise) ways that HCIs engage with public health (table 1 and online supplemental table 3A). We included all settings, countries and contexts, including any level of governance, not restricted to specific actors or stakeholders. We excluded abstract only, non-peer-reviewed studies, opinion pieces and broad discussions surrounding the topic. We searched five electronic databases from their inception to June 2021 (PubMed, EBSCO (CIINAHL, Econ Lit), OVID (MEDLINE, EMBASE, Global Health, PsycINFO, Health Management Information Consortium), Scopus and Web of Science) and handsearched reference sections of key papers.

**Table 1:** Study eligibility

Domain	Inclusion	Exclusion
Publication	Published in peer-reviewed journals, refer to humans	Abstract only, non-peer-reviewed
Language	English language	Non-English language
Field	All ‘harmful commodity industries’ (HCIs), including, but not limited to food, beverage, alcohol, tobacco and gambling, alone or in combination (or grouped as HCIs)	Does not include an HCI
Focus	Describing a mechanism or practice through which HCIs interact and engage with public health	Not describing ways in which HCIs and public health engage
Design	Frameworks, typologies, classifications, conceptualisations (including, eg, reviews, case studies, qualitative analyses, mapping)	Opinion pieces and broad discussions surrounding the topic (without an attempt to frame/collate/classify)

### Screening and selection

Database searches were managed using Covidence systematic review software (Veritas Health Innovation). Duplicates were removed. Using the study eligibility criteria (table 1), one reviewer (SM) screened all titles and abstracts and a selection of abstracts from this first screen were discussed with a second reviewer (EPV) to determine potential eligibility. Full texts from the agreed list of abstracts from this first screen were retrieved and screened independently, with reasons for exclusion tabulated and subsequently discussed to resolve disagreements (SM and EPV).

Dixon-Woods *et al*^40^ describe CIS as ‘interpretive’ because only relevant examples from the literature are synthesised. We included papers that presented additional novelty, presented a new framework, domain(s) or unique interaction(s), rather than including all of the relevant literature. Where multiple papers examined the same initiative and mechanisms, one was selected. We created a hierarchy to guide a systematic and purposive selection (online supplemental table 3B), using preidentified key papers, the emerging typology and principles of theoretical saturation.^45^

### Data extraction

With input from the wider authorship and public and practitioner collaborators, we created a guiding extraction frame^40^ to direct data extraction, with both inductive and deductive interpretation to capture emergent concepts from the literature. Data extraction included reference details (eg, authors, year, study type, setting, field, industry), framing (terminology, classification, clusters, including figures), interactions/activities (nature, type, actors) and any associated relevant examples or contextual information. Although a formal quality assessment or ‘risk of bias’ assessment did not apply to our research, we maintained an ongoing critical orientation to the material selected.^40^

### Data synthesis (typology development)

Guided by CIS principles,^40^ we synthesised key theoretical frameworks and taxonomies of previous efforts to describe, monitor and collate corporate interactions of industries, regarding public health. We gathered and coded fragments of text, grouped the codes into themes, used data charting,^47^ sifting and sorting, to simultaneously create and adapt the typology, organise higher-level categories and themes, and add further details based on the included studies.

### Review and analysis of the grey literature

We conducted a review and analysis of the grey literature, to provide contextual information, including what, why, who and how the HCI interactions potentially manifest at the LA level,^48^ through searching targeted websites, online resources, relevant publicly available grey information and consulting experts. We aimed to find further domains and representative documented case examples of these interactions at English LA level, rather than a comprehensive assessment.

We used the typology developed for the above review to direct our search of grey information in a targeted way and capture examples relating to English LA interactions with HCIs. The typology was reviewed (by SM, EPV and MW), refined in consultation with the wider authorship, public and practitioner collaborators, and translated into a practical list of terms to inform the search and a preliminary list of documents (online supplemental table 4). We applied similar methods to systematic review search strategies and handsearching grey literature.^49 50^ Potentially relevant records were ‘bookmarked’ in the web browser (Google Chrome), and we then searched within each bookmarked website. Searches continued within an a priori pragmatic time frame of 3 months to the point of informational and document saturation.^51^

Results of the searches were screened online, and relevant examples were documented in an Excel database, including the information source, industry, LA, interaction and any important contextual commentary. Using a CIS approach facilitated the highly iterative stance, allowing for ‘question modification’^40^ in response to the emergent findings, which altered and refined the typology.

## Results

**Figure 1:**
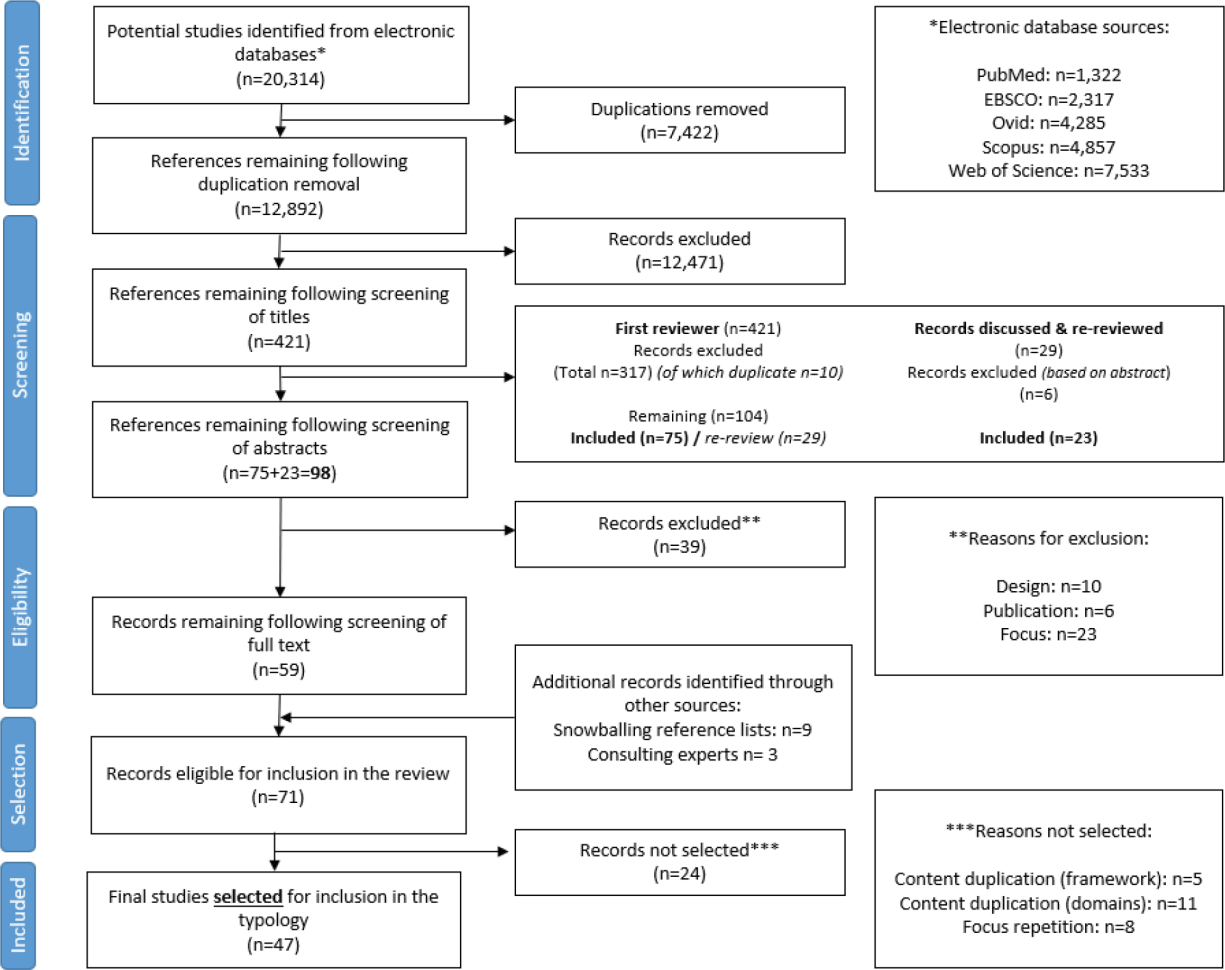
Study selection process (PRISMA flow diagram). PRISMA, Preferred Reporting Items for Systematic Reviews and Meta-Analyses.

The results of the identification, eligibility and selection process are shown in figure 1. Out of the 71 potentially eligible studies, we did not select 24 due to content duplication of: the framework (n=5); the domains (n=11); or the study focus (n=8) (online supplemental table 5). The retained 47 studies included reviews (n=19; 40%), qualitative analyses (n=8; 17%), essays (n=7; 15%), short pieces (n=5; 11%), case studies (n=4; 9%), concept mapping (n=2; 4%) and theory/tool development (n=2; 4%). HCIs in these papers included tobacco (n=10; 21%), food (n=10; 21%), baby food (n=1; 2%), non-specific ‘unhealthy commodity industries’ or private sector/corporations/industries harmful to health (n=13; 28%), gambling (n=5; 11%), alcohol (n=4; 9%) and a combination of two industry types (n=4; 9%). Full details are described in online supplemental table 6, including the 24 studies not selected (online supplemental table 5). Grey literature evidence included LA websites and reports, non-governmental organisations (NGOs), government documents, news and media reports, among other sources (online supplemental table 7).

### An overview of the context in the current literature

We did not identify any previous study that analysed interactions between HCIs and LAs. The conceptualisations, framing and language used to describe and understand the ways industry engages with public health was weighted towards the CDoH,^2 6 18 44 52–55^ corporate and commercial power,^1 11 13 56–59^ CPA,^23 46 60–67^ tactics^10 68^ or strategies^69–71^ and CSR^22 72–79^ literature. Studies were mostly in a broad context, such as global or population health settings, focused on international policy and examining transnational corporations. For example, exploring corporations’ commercial involvement in public health policy,^80^ or a framework and mechanisms of transnational corporations’ macrosocial and corporate tactics regarding population health and policy.^13^ Some studies focused on specific populations, industries, policies or activities, but remained in a similar lens, focusing on the CPA of industries.

### A proposed typology of possible interactions between HCIs and English LAS

Table 2 presents our proposed typology of the interactions between HCIs and English LAs. Three categories of potential interactions were developed, based on the medium through which the interactions take place: (1) direct involvement with the LA (governmental), (2) involvement through intermediaries (third parties) and (3) involvement through the local knowledge space. The typology expands on these three categories, with 10 themes describing the nature of interactions, encompassing over 50 potential interactions and illustrative examples.

**Table 2:** A typology of potential interactions between harmful commodity industries (HCIs) and local authorities (LAs) with illustrative examples in the English context

Themes	Interactions	Examples
**Category 1: involvement with the LA (governmental)**		
(1) Contractual, legal and regulatory	Commissioning (eg, service provision, industry organising an event LA funds) Licensing (Premises licences for alcohol/drinking establishments, fast food outlets and operating/premises/personal licences for gambling outlets) ‘Pouring rights’ contracts, franchises (eg, sales in a venue, local schools, hospitals) Marketing/advertising on the LA platform (LA-owned property/advertising spaces) Industry withdrawal/termination of local investments/contracts/resources/revenue if new local public health policies are introduced LA investments/business links/financial ties linked with industry and joint endeavours Litigate, sue, use court injunction or threaten—‘regulatory chill’, against LA policies, organisations or individuals Create loopholes in local-level laws, agreements and rights Illicit trade, price-fixing, bribery, grey market activity and corruption in the locality	Fast-food franchise in a hospital LA purchase of a retail park (including fast-food outlets) to generate income LA pension investment in Tobacco and HFSS LA investment in supermarket HFSS brand litigation case against LA that rejected planning for a new outlet development Appealing LA decisions to reject new planning applications for A5 hot food takeaways Seasonal HCI marketing/promotional activities on LA owned spaces
(2) Voluntary engagements	Public–private partnerships between industry and local government (eg, joint ventures, concessions, grants, project-financed structure, outsourcing, leasing) Cooperative arrangements (voluntary/coregulation/self-regulation, agreements and pledges) Direct involvement in policy making and the local public health agenda (eg, meetings, local stakeholder consultations, petitions, working/technical/advisory groups/boards) Provide input to local policy makers and attend workshops, and write/draft policies	Children Programme Primary Authority partnership between LAs and HFSS brands LAs outsourcing Public Service contracts to third sector bodies Voluntary LA joint project with HFSS food outlet to test a nudge intervention
(3) Other engagement strategies	Funding/financial incentives to local political parties, policy makers, legislators, groups (eg, donations, gifts) Local policy makers taking/movement across posts in the industry—the ‘revolving door’ Establish relationships and network with decision-makers and key governmental persons, including use of the internet and social platforms ‘journo-lobbying’, for wider access Employ adapted and reactive strategies and campaigns in response to local events/policy, such as shifting the corporation (jobs and consumers) to localities without restriction Delay implementations of regulation/intervention locally and limit liability Costs/consequences of policy compliance (all-encompassing local impact, for the LA, the local community), including time, money and business opportunities	LA and HCI memberships in the All-Party Parliamentary Group on a fit and healthy childhood (the APPG) LA awarding money to improve the appearance of a HFSS food outlet in the local area
**Category 2: involvement through intermediaries (third parties)**		
(1) Contractual	Cobranding initiatives in the locality Jobs and market created and contracted in the locality (eg, suppliers, construction) Local cause-related marketing (profit funds towards an LA project, refurbishment, resource)	Health campaign events cobranded with unhealthy beverage industry
(2) Voluntary engagements	Funding of NGOs that work with the LA (charities, SMEs) Support/sponsor locally (eg, health, arts, events, community-level initiatives, sport), contribute to/lead local programmes, information, curriculum, resources/technical support Donations to local groups/community ‘worthy causes’ (develop, renovate) Create local awareness groups, solution/therapy programmes, youth prevention	Community Alcohol Partnerships LA investment portfolios and commercial borrowing LA-HCI joint membership and funding on environmental, youth, food insecurity, neighbourhood schemes and community groups UK charity partnership with HFSS brand to support children’s educational programmes
(3) Other engagement strategies	Placement of industry-friendly persons/advocates within local key influential NGOs ‘Interlocking’ directorates/presence on board of directors, legal, PR/marketing firms and lobbyists across local industry · Establish relationships with key opinion leaders and spokespersons in the local area · Establish relationships with local media, PR associations, journalists, bloggers to communicate concerning LA issues Local ‘pan-industry’ groups, multistakeholder networks, think-tanks (joining local industry) Create local grassroots or fake—‘Astroturf’ organisations, front groups, forums	Research centres funded by HFSS industries HFSS food Franchisee maintaining close connections with LA and the local community Unhealthy beverage industry acquiring healthful brand and partnering with school campaigns in LA funded schools across the UK
**Category 3: involvement through the local knowledge space**		
(1) Influence local health messages	Fund and co-opt researchers, scientists, academics, chairs, university programmes, ghost writers, institutions, foundations, consultants and spokespersons on public health matters Maintain relationships with research funding councils to shape the research agenda (in terms of research questions and how to frame them, and priorities for funding) Participate/host scientific events, preconferences and side events (eg, briefings) Promotion of public health data and messages aligned with industry goals	Unhealthy beverage industry funding research foundations Unhealthy beverage industry membership in health research ‘clubs’ Conference on healthy eating and physical activity partnered with unhealthy beverage industry
(2) Challenge the local public health narrative	Undermine those promoting alternative public health viewpoints (LA advocates, organisations, experts and key local spokespersons) Reframe the LA intentions, create controversy (overprotective LA—‘nanny state’, coercive, commodity consumption is a personal choice/blame the victim) Create antagonisms/rivalry between LA departments and professionals Monitoring activities, operations and advocacy strategies of local public health functions Gather intelligence of public attitudes, policy development and key persons Advocate data favouring industry, and promote non-peer-reviewed, unpublished, or misleading/skewed information—‘junk science’ Manage information sources and availability of sources, through co-opted spokespersons Seek to control local public health-related research not aligned with industry arguments Change the frame of the LA narrative (emphasise doubt, disagreement, bias)	LA collaborative programme (JCDecaux UK and Outsmart) to ban HFSS food advertising from LA-owned advertising spaces
(3) CSR interactions (LA promotion of CSR)	LA promoting, endorsing, display support of initiatives with industry LA promoting industry local initiatives (eg, environmental—‘greenwashing’) Run campaigns with industry Fund public health services (eg, school/hospital related CSR)	LA endorsing a campaign, including explicit link with unhealthy beverage industry Unhealthy beverage industry and LA exclusive sponsorship of recycling bins in public spaces LA promoting fast-food ‘good times’ summer activities programme Industry primary sponsor of the Daily Mile
(4) CSR aligned with LA goals (HCIs presenting themselves as acting within the interests of LAs and communities)	Good traits of industry (individual and collective benefit/contributions to local area/society) Revenue generated from rents, taxes, business rates Draw on perceptions that industry expertise could reduce costs, raise quality, innovation, efficiency) Industry is responsible/part of the solution—solving/actions to address public health issues (including problems they create) · Shift responsibility away from HCI (focus on subpopulations, individual responsibility to consume according to industry recommendations, focus on other industries or events) Emphasising localism and ‘place narrative’ of the brand/industry, links with local roots, elite circles and cultural patronage Locality integration—‘localisation’, aligning industry products with LA area desire/demand Local workforce benefits (wages, health and safety, employee quality of life)	Gambling industry highlight £292 m in taxes in 2020 'Project open’ personalised business support from unhealthy beverage industry Tobacco industry campaigns against illicit trade, linking them with local police HFSS industry Foundation funding community events and activities in LA HFSS industry campaign to drive local purchasing, playing to their origins as a small business and high street partners Gambling industry highlights the employment of around 100 000 people in the UK Tobacco funding the Clean-up Britain campaign

Note: Some of the interactions may fall into more than one category or link with other themes (not mutually exclusive). Further information for each example interaction can be found in online supplemental table 7.

CSR, corporate social responsibility; HFSS, high in fat sugar and salt; LA, local authority; NGO, non-governmental organisations; PR, public relations; SME, small-to-medium-sized enterprise.

**Figure 2:**
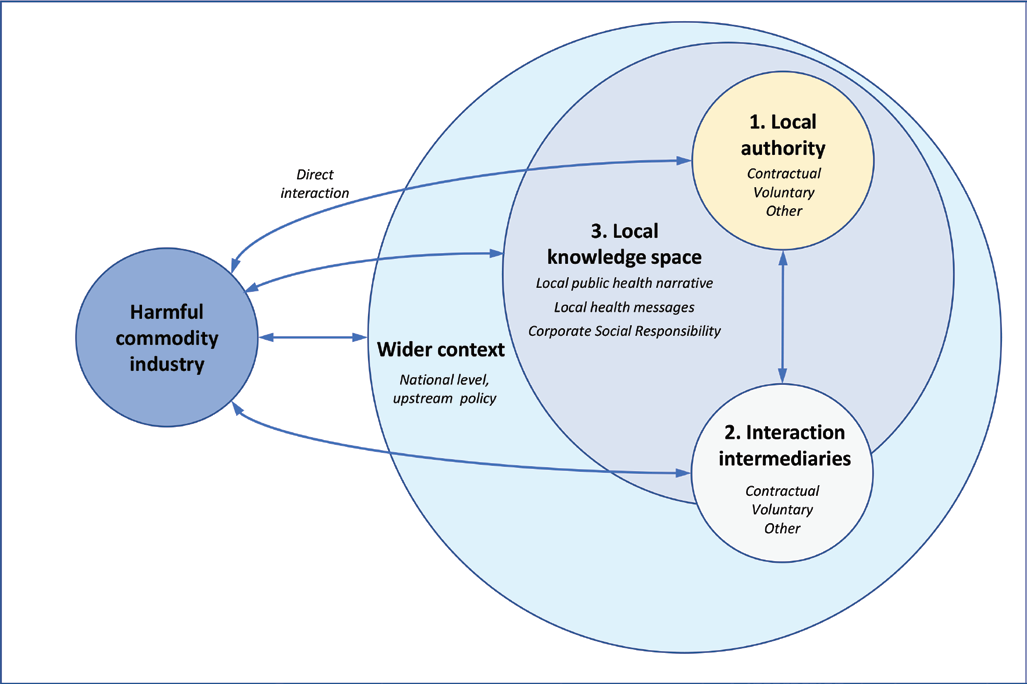
Visual representation of the interaction landscape.

Due to the complex and multifaceted nature and diverse presentation of potential interactions, the categories are not necessarily mutually exclusive, and some interactions cross multiple themes or categories. Also, other interactions take place within the wider context, at global and national levels, which shape and influence the local interaction landscape. Several interactions may also occur within the LA jurisdiction, but without explicit LA involvement, and some activities may remain within the traditional CPA lens. As a result, while many interactions and examples provided are specific and clear, we acknowledge that other, wider interactions (eg, with International and National bodies, NGOs, research councils) may be less clear, but have a significant presence and implications across multiple LAs. Figure 2 offers a visual representation of the typology and wider components contributing to the interaction landscape.

#### Category 1: involvement with the LA (governmental)

We identified three themes to summarise the ways in which HCIs and LAs directly interact with each other. The first theme includes contractual, legal and regulatory interactions involving formal arrangements between the two parties. These arrangements potentially involve monetary exchange, for example, licensing and registration of alcohol or drinking establishments, food outlets and gambling venues. Other interactions include business links, such as LA investments in industry, and joint endeavours in the LA jurisdiction. Legal interactions include direct litigation against a decision and the use of the legal system to threaten or contend LA policies, organisations, and individuals, stretch local legal boundaries or threaten legal outcomes. The second theme involves voluntary engagements between industry and LAs, and the most prominent examples include public–private partnerships, followed by cooperative arrangements (such as voluntary agreements and pledges). In addition, interactions include joint attendance in meetings and working groups, and voluntary input to policy makers in, for example, writing policies. The third theme includes all other engagement strategies. These interactions include incentivising career movement across public and private posts (‘revolving door’ arrangements), and relationship or network building. In addition, reactive strategies and responses to LA policies and decisions, and creating or emphasising disincentives, when arrangements are disagreeable.

#### Category 2: involvement through intermediaries (third parties)

The second category of interactions between HCIs and LAs specifically involves intermediaries to which we identified the same three themes. The first theme, contractual interactions, again mostly involve monetary exchange or shared financial interests, such as funding an LA project or services, cobranding in the LA jurisdiction or cause-related marketing. The second theme, voluntary interactions, include industry strategic support of local NGOs such as small-medium enterprises and charities, through partnering, funding and joint projects. These interactions often involve supporting local community ‘worthy causes’, claim to act in the interests of communities and offer solutions for LA priorities and community needs. For example, during the COVID-19 pandemic, larger industries provided funding smaller local businesses struggling with reduced demand.^81^ The third theme, other engagement strategies, closely aligns HCIs with key stakeholders in the local area, creating HCI support and networks, and broadening the interaction landscape. For example, relationships with local key opinion leaders, business links such as financial ties across industry (eg, investments and shares), or other interlocking features, such as shared directorates, public relations (PR), legal or marketing firms across industry. In addition, the media forms a vital platform as an interface between industry, LA and the wider public, providing a mode of indirect communication, through local media, PR associations, journalists and bloggers, providing access to local councillors, stakeholders or specific communities.

#### Category 3: involvement through the local knowledge space

Interactions most commonly identified from our searches are those involving the local knowledge space (the local area in which the LA has jurisdiction), through influencing local health messages, challenging the local public health narrative and CSR strategies. It is important to acknowledge that the wider, national-level knowledge space also influences the local knowledge space. First, HCIs interact with public health messages which influence the local knowledge space, for example, funding research activities and securing relationships with key researchers, to undermine, reframe and promote messages reflecting their goals and positionality. Other interactions include undermining key spokespersons, reframing LA intentions and creating or emphasising antagonisms. HCI CSR strategies could serve as an interaction interface through LA promotion of CSR, and CSR aligned with LA goals. Interactions could include the local economy (business rates, taxes, rents), employment opportunities in the locality (eg, suppliers, distribution, construction) or the local community (support, sponsorship, building/developing) among a plethora of other interactions involving local areas, the local working and living environment, extending from local schools to environmental initiatives (eg, sustainability, pollution abatement). These CSR interactions form part of a strategy for HCIs to present themselves as acting within public interests, to shift the blame, whitewash tarnished reputations and enhance the normalisation of products known to be harmful to health.

## Discussion

### Summary of main findings

To our knowledge, this is the first study to develop a typology of the potential interactions between HCIs and local government, with case examples drawn from England. Drawing on previous published frameworks and conceptualisations, and grey literature, our typology classifies potential interactions according to the medium through which they take place. Interactions occur both directly with the LA and indirectly through intermediaries by contractual, voluntary and influential means, and via the local knowledge space, involving local health messages, the local public health narrative and CSR. Our perspective and findings acknowledge the wider CDoH environment, and upstream contextual factors, which shape the interaction landscape at a local level.

Given the extent of potential interactions, we suggest that the interactions between HCIs and local government may have a significant role in shaping local environments in which people work and live, and thus health behaviours and associated outcomes at a population level. Our conceptualisation of interactions acknowledges the LA perspective, in which working with the private sector generally is often seen as acceptable by LAs to execute functions to enhance local society, in and out of the public health domain (eg, planning, community development, transport). The proposed typology is not definitive, and could be enhanced with further evidence, synthesis and future research.

### Strengths and limitations

A major strength of this study is that it proposes a novel and detailed typology with methodological rigour, that identifies ways HCIs and LAs interact, which may be used by LAs to assess their own practices. To the best of our knowledge, this is the first framework to consider HCI interactions at an LA level. Drawn on well-established CDoH theories and frameworks, our typology offers new utility and applicability for research and practice. The provided typology should enable further study of these interactions and develop pragmatic ways in which LAs and HCIs can interact, to maximise population health interests.

This study has some limitations. First, we only used five databases for our literature search, which although in combination offered a diverse range of literature, may not fully depict the business relations documented in the wider literature. Although this paper did not aim to review all the relevant work, some important, related work, may not have been included. The lens, of studying ‘interactions’ with HCIs, has not been previously adopted and previous literature, including the underpinning theory, methods and illustrative cases, is mostly skewed towards a CPA lens. In addition, despite the aim to adopt a lens that reflects the LA viewpoint besides the public health perspective, we acknowledge the potential for bias in our interpretation.

Although we aimed to capture local level interactions, we acknowledge that there are other influences, including at national and international level, which manifest locally but are not captured in detail in this manuscript, due to the specific focus on LA levers. We acknowledge that by applying our typology to the English LA context, our examples may be most aligned with similar political systems in HIC settings. However, transnational harmful commodity organisations operate on global markets and their approaches share commonalities across both HIC and LMIC settings, but this has not been explored at an LA level and warrants further research. The reliance on publicly available information to gain the LA context also has limitations. Some of the interactions lack documented evidence and are dominated by opinion-based and anecdotal evidence, and hidden, invisible, informal and some indirect interactions were not captured.

### Situating the typology within the wider literature

While previous efforts to describe and monitor the practices of HCIs have focused on CPA efforts and in a wider context, we focus on interaction, specifically in the English LA context. We have not attempted to describe or define the CDoH or CPA, or the broad dynamics that constitute elements previously comprehensively synthesised.^52 82^ However, the current typology is derived from this literature, most prominently utilising aspects of CPA formerly identified.^46 63^ Mialon *et al*^46^ developed a step-by-step approach to monitor the CPA of the food industry within countries. In addition, the ‘Corporate Permeation Index’^83^ quantifies the penetration of corporations in a given country. Similar methods could be developed to examine and monitor interactions between HCIs and LAs, using the current typology.

The lens and typology we present recognises that interactions between HCIs and LAs are fundamental for LA functioning. Overwhelming evidence^1 2 6–15 84^ opposes HCI involvement, and stresses the significant harm to the health of the public from public health and corporate HCI partnerships, with limited benefits.^85^ Contrasting arguments suggest that the benefits of commercial influence (eg, economic growth) outweighs adverse health outcomes,^86^ and private sector interactions more generally are viewed as potentially mutually beneficial to the wider, non-public health stakeholder.^18 86 87^ While acknowledging that population health improvement may necessitate interaction with the private sector,^18^ the wider long-term harmful effects of HCI interactions may not be adequately considered and accounted for, necessitating the application of criteria, conditions and safeguards to govern the commercial influences on population health. Further, the significance of the problem is likely to continue as local government financing constrictions prevail (UK and internationally) due to COVID-19 pressure, requiring more local funding activities, including through interactions with HCIs. Thus, our analysis suggests there is the need and opportunity to develop LA guidance to support local decision making that considers the population health impacts and minimises the health and reputational risks of HCI interactions.

In recognising the wider context in which our typology is situated, we echo the importance of wider interconnected elements and need for interventions across whole systems at the LA level.^88^ LAs hold unique and substantial powers to shape local environments through various mechanisms. Despite LAs having powers to govern HCI interactions and protect public health, we found that the extent to which LAs use their levers varied. For example, while some LAs have enacted commercial policies to restrict interactions such as those mirroring the TFL junk food advertisement ban, other interaction examples specifically demonstrate LAs seeking out commercial opportunities with HCIs, enabling advertisement and sponsorship of their products in the local area. Recent work^89^ reviewed LAs ability to contribute to the change process for healthy food systems and environments and stressed the need to empower LAs. A lack of LA empowerment or involvement could, in part, explain several of the identified potentially harmful interactions, such as, licensing and planning. A recent census study^90^ across English LAs explored takeaway food outlet planning and regulation. The authors observed just 56 of 325 LAs had health-specific planning criteria, compared with 80% non-health (eg, litter, anti-social behaviour), owing to the acceptability and probability of challenge.^90^ It is important that considerations for health should be embedded across all LA functions and decisions, as existing beliefs may dichotomise LA roles and functions into those which are health focused, and those which are not.^91^

LAs have proven capacity to take effective action, advance population health goals and overcome the CDoH,^89^ including managing the built environment,^90^ yet, more often, intervention design prioritises avoiding potential confrontation or dispute (eg, improving the healthfulness of takeaway outlets).^92^ Recent work by Mialon *et al*^93^ identified how Freedom of Information (FoI) disclosure logs, and disclosures of conflicts of interests have been adopted in the UK as mechanisms to monitor and address the influence of corporations on population health. Yet, their findings are at national, regional and global levels, without LA level evidence.^93^ LAs, through legislative and regulatory powers, can make significant and meaningful changes to business activities, structures, rules, norms and practices.^94^ Developing guidance and standards for interactions with HCIs could increase transparency and empower LAs to assess potential health impacts to the local population as well as possibilities of associated reputational risks.

### Research implications

The proposed typology of interactions presented can now be used to systematically explore further opportunities to add new interactions, and tailor the manifestation of interactions in both similar and distal contexts. The typology is not definitive but is a conceptual starting point to be modified and built on. Future work to supplement missing information could increase the validity, for example, through wider sharing of the typology and surveys to build consensus. Interviews with key stakeholders, such as LA actors, third party organisations and public representatives could describe their positionality, interactions used and outcomes. Involving stakeholders most affected is warranted to incentivise and empower policy makers to act, which otherwise presents a challenge to the acceptability and practical usability of the research. In addition, this would assist in identifying interactions that may not currently be evidenced in the public domain.

The typology was informed by international literature and translated to a local-level context. While we acknowledge the findings may be most aligned with similar political systems in high-income country settings, we suggest that the ‘interaction’ lens, within a local context, has the potential to be applied more generally. Further research is needed which accounts for the wider socioeconomic, and the importance of political economy and governance context, on HCI–LA interactions, to enhance the practical applicability of adopting this Framework.

We do not attempt to quantify the extent to which the interactions are attributed to outcomes, such as local NCD prevalence, health inequalities, commercial and wider public implications. Outcomes of the interactions should be assessed, to understand who benefits and the net consequences, for the industries, LAs and local communities involved. In addition, interactions between LAs and other industries (eg, coal, oil and gas) are also of public health concern and future work could extend the concepts described in this paper, across other HCIs.

### Policy and practice implications

To make meaningful improvements in health, future work could build on these findings to develop interventions and practical tools for specific actions that LAs can take. Standards could be adapted and applied to govern local interactions, such as previous work extending and tailoring the use of the ‘MPOWERR’ tool^89^ covering marketing, advertising, sponsorship and planning provisions. Guidance that incorporates an LA perspective, could ensure that LAs interact with HCIs in a way that optimises business opportunities while protecting population health and avoiding reputational risks.

## Conclusion

This study describes the development of a typology, drawn from a synthesis of the well-established academic literature in combination with local-level evidence. Our typology provides an overview of the potential interactions between HCIs and LAs. The typology can facilitate new understanding that explores the LA perspective of interactions with HCIs, to support LA policy and decision-makers, in and outside of public health. Furthermore, the typology should serve as a platform for future research to build on, as a practical approach to curtail the CDoH-NCD pandemic, and further supplement national level NCD policy actions (eg, obesity strategy), by focusing on HCI and LA interactions.

## Data Availability

Data are available on reasonable request. No data are available.
