# Supplementary material for: Typology of how ‘harmful commodity industries’ interact with local governments in England: a critical interpretive synthesis
Source: BMJ Glob Health. 2023 Jan 23;8(1):e010216. doi: 10.1136/bmjgh-2022-010216 (PMC9872461; doi:10.1136/bmjgh-2022-010216)
Supplement: Supplementary data [file bmjgh-2022-010216supp001.pdf]

Online Supplementary material

Article title: Typology of how 'harmful commodity industries' interact with local governments in England: a critical interpretive synthesis

Corresponding author: Dr Sarah McKevitt, Public Health Policy Evaluation Unit, School of Public Health, Imperial College London

s.mckevitt@imperial.ac.uk

Contents

1. Adapted PRISMA-ScR checklist
2. Review search strategy
3. Study eligibility and selection
4. Review and analysis of grey literature
5. Studies not selected for inclusion
6. Description of included studies
7. List of example interactions

Supplementary Table 1. ADAPTED Preferred Reporting Items for Systematic reviews and Meta-Analyses extension for Scoping Reviews (*adapted* PRISMA-ScR) Checklist

SECTION	#	Adapted PRISMA-ScR CHECKLIST ITEM	PAGE
**ABSTRACT**			
Summary	1	Provide a structured summary that includes (as applicable): background, objectives, eligibility criteria, sources of evidence, charting methods, results, and conclusions that relate to the objectives.	3
**INTRODUCTION**			
Rationale	2	Describe the rationale for the review in the context of what is already known. Explain why the review questions/objectives lend themselves to the approach.	5
Objectives	3	Provide an explicit statement of the questions and objectives being addressed with reference to their key elements (e.g., population, concepts, and context) or other relevant key elements used to conceptualize the objectives.	6
**METHODS**			
Protocol and registration	4	Indicate whether a review protocol exists; state if and where it can be accessed (e.g., a Web address); and if available, provide registration (number).	6
Eligibility criteria	5	Specify characteristics of the sources of evidence used as eligibility criteria (e.g., years considered, language, and publication status), and provide a rationale.	6-7
Information sources	6	Describe all information sources in the search (e.g., databases with dates of coverage and additional sources), as well as the date of the most recent search.	7
Search	7	Present the full electronic search strategy for at least 1 database, including any limits used, such that it could be repeated.	Sup3
Selection	8	State the process for selecting sources of evidence (screening and eligibility)	7-8
Data charting	9	Describe the methods of charting data from the included sources of evidence	8
Data items	10	List and define all data variables sought and any assumptions and simplifications	8
Critical appraisal	11	If appropriate, provide a rationale for conducting a critical appraisal of included sources of evidence (rationale why not)	8
Synthesis	12	Describe the methods of handling and summarizing the data	8-9
**RESULTS**			
Study selection	13	Give numbers of sources screened, assessed for eligibility, and included in the review, with reasons for exclusions at each stage, ideally using a flow diagram.	9
Study characteristics	14	For each source of evidence, present characteristics and provide the citations.	Sup5 &7
Results	15	For each included source of evidence, present the relevant data that were charted that relate to the review questions and objectives.	13-15
Synthesis	16	Summarize results as they relate to the review questions and objectives.	10-12
**DISCUSSION**			
Summary of evidence	17	Summarize the main results (including an overview of concepts, themes, and types of evidence available), link to the review questions and objectives, and relevance to key groups.	16
Limitations	18	Discuss the limitations of the review process.	16
Conclusions	19	Provide a general interpretation of the results with respect to the review questions and objectives, as well as potential implications and/or next steps.	17-18
**FUNDING**			
Funding	20	Describe sources of funding for the included sources of evidence, as well as sources of funding for the scoping review. Describe the role of the funders.	19

JBI = Joanna Briggs Institute; PRISMA-ScR = Preferred Reporting Items for Systematic reviews and Meta-Analyses extension for Scoping Reviews; SUP(n) = supplementary file number. *The frameworks by Arksey and O'Malley (6) and Levac and colleagues (7) and the JBI guidance (4, 5) refer to the process of data extraction in a scoping review as data charting. *Adapted From:* Tricco AC, Lillie E, Zarin W, O'Brien KK, Colquhoun H, Levac D, et al. PRISMA Extension for Scoping Reviews (PRISMAScR): Checklist and Explanation. Ann Intern Med. 2018;169:467–473. doi: 10.7326/M18-0850.

Supplementary Table 2. Review search strategy

2. A) Search topics, terms, and database logic

Date of search 17/06/21

Topic	Ref	Terms
**Environment**	#1	**TITLE** "commercial determinant*" OR "commercial involve*" OR "commercial epidemic" OR "commercial pandemic" OR "macrosocial determinant*" OR "corporate determinant*" OR "corporate involve*" OR "corporate epidemic" OR "corporate pandemic" OR "industry determinant*" OR "industrial determinant*" OR "industrial involve*" OR "industry involve*" OR "industry epidemic" OR "industrial pandemic" OR "unhealthy commodit*" OR "harmful commodit*" OR corporatology
**Industry**	#2	**TITLE** (Food OR Sugar OR Transfats OR “Ultra-process*” OR Alcohol OR Drink* OR Beverage OR Tobacco OR Cigarette OR Nicotine OR Smok* OR Gambl* OR Commerc* OR “For-profit” OR Transnational OR Private)<br><br>AND **TITLE** (Industr* OR Big OR Corporat* OR Determinant* OR Compan* OR Firm* OR Manufactur* OR Organisation* OR Organization* OR Sector* OR Environment)
**Interaction**	#3	**TOPIC** (Activit* OR Advertis* OR Advis* OR Agreement* OR Capture OR CDOH OR citizenship OR "Civil society capture" OR Collaborat* OR Communicat* OR "Concerned citizenship" OR Conflict* OR "Constituency building" OR Cooperat* OR Coopt OR “co-opt” OR "Corporate social responsibility" OR "Corporate-state" OR "Corporation-induced" OR CSR OR Determinant* OR Deregulat* OR Donat* OR "Financial incent*" OR "Framing dominant" OR Fund* OR Health OR Influenc* OR Interact* OR Invest OR Invested OR Investment OR Investor OR Investing OR Involve* OR Joint OR Lobby* OR Neoliberal OR “Neo-liberal” OR "Non-market strateg*" OR partner* OR Philanthrop* OR Playbook OR "Policy capture" OR Power OR Practice* OR "Preference shaping" OR “Private-public” OR “Public-private” OR Relation* OR Spinning OR Sponsor* OR “State-corporate” OR Strateg* OR Support* OR Tactic OR Tie)
**Framework**	#4	**TOPIC** (Appraisal OR Categori* OR Classif* OR Codif* OR Conceptuali* OR Framework OR Mechanism* OR Model OR Schema OR Taxonom* OR Theory OR Trend* OR Typif* OR Typolog* OR Overview)
	#5	**#2 AND #3 AND #4**
	#6	**#1 OR #5**
**Additional NOT filters**[1]	#7	**TITLE** (Healthcare OR Hospital OR Gene OR Genom* OR Genetic* OR Genotyp* OR Trial OR Intervention)
	#8	**#6 NOT #7**
**Filters:**		**English language, de-duplicate (remove duplicates)**[2]**.**
		**Human filter (see table below)**

[1]*only if needed after preliminary screen.* [2]*Remove duplicates within and across databases.*

**Database specific logic**				
**PubMed**	**Scopus**	**Web of science**	**Ebsco**	**Ovid**
	Pre/2	Near/2	N2	Adj2
			Separate title / abs (no topic)	Separate title / abs (no topic)
				Automatic phrasing (no “”)
**NOT ("Animals"[Mesh] or animal)**	NOT (INDEXTERMS(animals OR animal)			NOT (exp animal/ or animal)

2. B) Example search strategy string: PubMed

(("commercial determinant*"[Title] OR "commercial involve*"[Title] OR "commercial epidemic"[Title] OR "commercial pandemic"[Title] OR "macrosocial determinant*"[Title] OR "corporate determinant*"[Title] OR "corporate involve*"[Title] OR "corporate epidemic"[Title] OR "corporate pandemic"[Title] OR "industry determinant*"[Title] OR "industrial determinant*"[Title] OR "industrial involve*"[Title] OR "industry involve*"[Title] OR "industry epidemic"[Title] OR "industrial pandemic"[Title] OR "unhealthy commodit*"[Title] OR "harmful commodit*"[Title] OR corporatology[Title] AND (english[Filter])) OR ((((Food[Title] OR Sugar[Title] OR Transfats[Title] OR "Ultra-process*"[Title] OR Alcohol[Title] OR Drink*[Title] OR Beverage[Title] OR Tobacco[Title] OR Cigarette[Title] OR Nicotine[Title] OR Smok*[Title] OR Gambl*[Title] OR Commerc*[Title] OR "For-profit"[Title] OR Transnational[Title] OR Private[Title]) AND (Industr*[Title] OR Big[Title] OR Corporat*[Title] OR Determinant*[Title] OR Compan*[Title] OR Firm*[Title] OR Manufactur*[Title] OR Organisation*[Title] OR Organization*[Title] OR Sector*[Title] OR Environment[Title]) AND (english[Filter])) AND (Activit*[Title/Abstract] OR Advertis*[Title/Abstract] OR Advis*[Title/Abstract] OR Agreement*[Title/Abstract] OR Capture[Title/Abstract] OR CDOH[Title/Abstract] OR citizenship[Title/Abstract] OR "Civil society capture"[Title/Abstract] OR Collaborat*[Title/Abstract] OR Communicat*[Title/Abstract] OR "Concerned citizenship"[Title/Abstract] OR Conflict*[Title/Abstract] "Constituency building"[Title/Abstract] OR Cooperat*[Title/Abstract] OR Coopt[Title/Abstract] OR "co-opt"[Title/Abstract] OR "Corporate social responsibility"[Title/Abstract] OR "Corporate-state"[Title/Abstract] OR "Corporation-induced"[Title/Abstract] OR CSR[Title/Abstract] OR Determinant*[Title/Abstract] OR Deregulat*[Title/Abstract] OR Donat*[Title/Abstract] OR "Financial incent*"[Title/Abstract] OR "Framing dominant"[Title/Abstract] OR Fund*[Title/Abstract] OR Health[Title/Abstract] OR Influenc*[Title/Abstract] OR Interact*[Title/Abstract] OR Invest[Title/Abstract] OR Invested[Title/Abstract] OR Investment[Title/Abstract] OR Investor[Title/Abstract] OR Investing[Title/Abstract] OR Involve*[Title/Abstract] OR Joint[Title/Abstract] OR Lobby*[Title/Abstract] OR Neoliberal[Title/Abstract] OR "Neo-liberal"[Title/Abstract] OR "Non-market strateg*"[Title/Abstract] OR partner*[Title/Abstract] OR Philanthrop*[Title/Abstract] OR Playbook[Title/Abstract] OR "Policy capture"[Title/Abstract] OR Power[Title/Abstract] OR Practice*[Title/Abstract] OR "Preference shaping"[Title/Abstract] OR "Private-public"[Title/Abstract] OR "Public-private"[Title/Abstract] OR Relation*[Title/Abstract] OR Spinning[Title/Abstract] OR Sponsor*[Title/Abstract] OR "State-corporate"[Title/Abstract] OR Strateg*[Title/Abstract] OR Support*[Title/Abstract] OR Tactic[Title/Abstract] OR Tie[Title/Abstract] AND (english[Filter]))) AND (Appraisal[Title/Abstract] OR Categori*[Title/Abstract] OR Classif*[Title/Abstract] OR Codif*[Title/Abstract] OR Conceptuali*[Title/Abstract] OR Framework[Title/Abstract] OR Mechanism*[Title/Abstract] OR Model[Title/Abstract] OR Schema[Title/Abstract] OR Taxonom*[Title/Abstract] OR Theory[Title/Abstract] OR Trend*[Title/Abstract] OR Typif*[Title/Abstract] OR Typolog*[Title/Abstract] OR Overview[Title/Abstract] AND (english[Filter])) AND (english[Filter])) AND (english[Filter])) NOT (Healthcare[Title] OR Hospital[Title] OR Gene[Title] OR Genom*[Title] OR Genetic*[Title] OR Genotyp*[Title] OR Trial[Title] OR Intervention[Title] AND (english[Filter])) AND ((humans[Filter]) AND (english[Filter]))

2. C) Database and additional search results (numbers)

**Database**	**Number of references**
PubMed	1,322
EBSCO (CINAHL, Econ Lit)	2,317
OVID (MEDLINE, EMBASE, Global Health, PsycInfo, HMIC)	4,285
Scopus	4,857
Web of Science	7,533
Duplications removed	*-7,422*
Screening process removed	*-12,833*
Snowballing reference lists	9
Consulting experts	3
Selection process removed	*-24*
Total	47

Supplementary Table 3. Study eligibility and selection

Stage 1. Study eligibility

3. A) Is the study eligible to be included in the final review? (brackets = reason for exclusion)

**Eligibility criteria**	Yes	Unclear	No
1. Is the study full text and peer reviewed? (publication)			
2. Is the study in English language? (language)			
3. Does the study focus on <u>any</u> type of harmful industry explicitly, such as one or more of; food, beverage, alcohol, tobacco or gambling (alone or in combination)? (field)<br>*e.g. we will not include pharmacological industry unless this is combined with an eligible industry in the same study.*			
4. Is the study aim to capture industry interactions/activity? (focus)			
5. Primary outcomes (design)<br>a) Does the study include a framework / classification / description of industry interactions / activities			
Final decision on study eligibility	Eligible	Unclear	Exclude

Stage 2. Only if the study is Eligible in Stage 1.

3. B) Is the study selected for the review?

**Selection criteria**		Yes	Unclear	No
1. Is the framework new / novel				
2. Or what framework does this study build upon (added novelty)	*Detail*			
3. Or other reason for selection	*Detail*			
Final decision on study selection		Selected	Unclear	Exclude

Only studies which (1) meet the study eligibility in Stage 1, and (2) fulfil one of the selection criteria will be selected.

Supplementary Table 4. Review and analysis of grey literature

4. A) Grey information search strategies

**Strategy 1**	Targeted website searches (local authority [LA] documents)
**Strategy 2**	Customized searches of webpages and other online resources
**Strategy 3**	Relevant publicly available grey information (including media)
**Strategy 4**	Consultation with experts including practitioners working in local authorities (LAs)

4. B) Preliminary list of documents

Budget announcements
Business rates
Commercial policies
Committee meeting minutes
Concerns / praises raised by community
Contracts, tenders
Design papers
Funding streams
General codes of conduct / legal frameworks / rules for interactions
Grant requirements
Impact analyses in local authority portfolio
Joint Strategic Needs Assessment
Licensing (licensing act changes)
Meeting minutes (funding committee, council meeting of chief executives, GLA)
Pension funding
Planning permissions
Position statements
Procurement contracts
Published statements / policies
Registers of financial interests
Reports
Standards
Sub-contracts
Treasury policy documents
Voluntary initiatives
Websites

4. C) Document key search terms (extracted from Part 1 results)

**Engagement**	**Responsibility**	**Presence**	**Actors**
**Legislation**	Business rate	Petition	Shareholders
**Acquisition**	Donate	Advertise	Business
**Agreement**	Fund	Brand	Commercial
**Contract**	Investment	Campaign	Corporation
**Co-regulation**	License	Consultation	Franchise
**Joint project**	Market	Forum	Industry
**Joint venture**	Pouring right	Initiative	Organisation
**Merger**	Rent	Promote	Stakeholder
**Partnerships**	Resources	Roundtable	Company
**Pledge**	Shares	Think-tank	Private sector
**Policy**	Sponsor	Workshop	
**Self-regulation**	Support	Meeting	

Supplementary Table 5. Studies eligible but not selected for inclusion in the current study (n=24)

Primary author	Year	Title	Reasons for not selecting[1]
**Baum**	2016	Assessing the health impact of transnational corporations: its importance and a framework.	Focus
**Brown**	2019	Legislative Capture: A Critical Consideration in the Commercial Determinants of Public Health.	Focus
**De Lacy-Vawdon**	2020	Defining the commercial determinants of health: a systematic review.	Content duplication (framework)
**Fooks**	2011	Corporate social responsibility and access to policy Elites: an analysis of tobacco industry documents.	Focus
**Freudenberg**	2012	The manufacture of lifestyle: the role of corporations in unhealthy living	Content duplication (domains)
**Gilmore**	2011	Public health, corporations and the New Responsibility Deal: promoting partnerships with vectors of disease?	Content duplication (domains)
**Hawkins**	2018	Reassessing policy paradigms: A comparison of the global tobacco and alcohol industries.	Content duplication (domains)
**Hill**	2020	'As Long as It Comes off as a Cigarette Ad, Not a Civil Rights Message': Gender, Inequality and the Commercial Determinants of Health.	Focus
**Holden**	2009	Corporate Power and Social Policy: The Political Economy of the Transnational Tobacco Companies.	Content duplication (domains)
**Jernigan**	2009	The global alcohol industry: An overview.	Content duplication (domains)
**Kadandale**	2019	The palm oil industry and non-communicable diseases.	Content duplication (framework)
**Lacy-Nichols**	2021	Power and the commercial determinants of health: ideas for a research agenda.	Content duplication (domains)
**Maani**	2020	Bringing the commercial determinants of health out of the shadows: a review of how the commercial determinants are represented in conceptual frameworks.	Focus or content duplication (domains)
**Ndebele**	2020	Commercial determinants of health: an ethical exploration.	Focus
**Paixao**	2019	Help or Hindrance? The Alcohol Industry and Alcohol Control in Portugal	Content duplication (framework)
**Richards**	2015	Corporate Social Responsibility programs of Big Food in Australia: a content analysis of industry documents.	Content duplication (domains)
**Sacks**	2013	A proposed approach to monitor private-sector policies and practices related to food environments, obesity and non-communicable disease prevention.	Focus
**Saloojee**	2000	Tobacco industry tactics for resisting public policy on health	Content duplication (domains)
**Swinburn**	2013	Monitoring and benchmarking government policies and actions to improve the healthiness of food environments: a proposed Government Healthy Food Environment Policy Index.	Focus
**Tanrikulu**	2020	Corporate political activity of the baby food industry: the example of Nestle in the United States of America.	Focus
**Tselengidis**	2019	Lobbying against sugar taxation in the European Union: Analysing the lobbying arguments and tactics of stakeholders in the food and drink industries.	Content duplication (domains)
**Vandenbrink**	2020	Strategies used by the Canadian food and beverage industry to influence food and nutrition policies.	Content duplication (framework)
**Weishaar**	2012	Global health governance and the commercial sector: a documentary analysis of tobacco company strategies to influence the WHO framework convention on tobacco control.	Content duplication (domains)
**Williams**	2015	Conceptualising the Commercial Determinants of Health Using a Power Lens: A Review and Synthesis of Existing Frameworks	Content duplication (framework)

[1]*Reasons correspond with those listed in the PRISMA flow diagram.*

Supplementary Table 6. Description of included studies (n=47)

First author	Year	Title	Design	Sources	Industry	Framework	Context	Key term	Figure extracted
**Amul**	2021	A Systematic Review of Tobacco Industry Tactics in Southeast Asia: Lessons for Other Low- And Middle-Income Regions	Review	Literature	Tobacco	Not specified	LMICs	CSR	Yes
**Anaf**	2017	Assessing the health impact of transnational corporations: a case study on McDonald's Australia.	Case study	Document analysis	Food	CHIA	Australia	Globalisation	Yes
**Babor**	2013	Public health, academic medicine, and the alcohol industry's corporate social responsibility activities.	Review	Literature	Alcohol	Health-related CSR	Academia, public health science	CSR	No
**Baker**	2021	Globalization, first-foods systems transformations and corporate power: a synthesis of literature and data on the market and political practices of the transnational baby food industry	Review	Literature and industry evidence	Baby food	Combination of CDOH, power, lobbying, corporate science	Global baby food	CDOH, power	Yes
**Barraclough**	2008	A grim contradiction: the practice and consequences of corporate social responsibility by British American Tobacco in Malaysia.	Review	Document and literature	Tobacco	CSR	Malaysia	CSR	No
**Bero**	2003	Implications of the Tobacco Industry Documents for Public Health and Policy	Review	Document analysis	Tobacco	Not specified	Tobacco generally	Tactics	No
**Bunn**	2020	The growth of sports betting in Malawi: corporate strategies, public space and public health.	Media analysis (qualitative)	News and advertising	Gambling	Not specified	Malawi	Strategies	No
**Buse**	2017	Healthy people and healthy profits? Elaborating a conceptual framework for governing the commercial determinants of non-communicable diseases and identifying options for reducing risk exposure.	Review	Literature	UP-food, beverage, alcohol, tobacco	Buse & Naylor	Global health governance	Commercial determinants of ill-health	No
**Campbell**	2020	How are frames generated? Insights from the industry lobby against the sugar tax in Ireland.	Review	Document analysis	Food	Framing as CPA	Ireland Sugar sweetened beverage tax	CPA, framing	Yes

**Canella**	2015	Food and beverage industries' participation in health scientific events: considerations on conflicts of interest.	Case study	Event congress	Food & beverage	Not specified	Scientific events	COI	No
**Cavalcanti**	2019	Benchmarking food and beverage company investment in healthful eating and active living initiatives	Case studies	Industry funded initiatives	Food & beverage	Commitment to healthy Communities Initiative	Corporate investment in community health	Philanthropy, voluntary initiatives, CSR	Yes
**Chavez-Ugalde**	2021	Conceptualizing the commercial determinants of dietary behaviours associated with obesity: A systematic review using principles from critical interpretative synthesis	Review	Literature	Food	Commercial determinants of dietary behaviours and obesity	Obesity and food	CDOH	Yes
**Clapp**	2017	Big Food, Nutritionism, and Corporate Power	Review	Literature	Food	Corporate power in food system	Big food generally	Power	Yes
**Eastmure**	2020	Non-market strategy as a framework for exploring commercial involvement in health policy: A primer.	Essay	Literature	Corporations	Non-market strategy as a conceptual lens	Public health generally	Non-market strategy	No
**Fooks**	2013	Corporate philanthropy, political influence and health policy	Document analysis (qualitative)	Industry documents, dialogue reports	Tobacco	Built on BAT CSR	Philanthropy and charitable giving	Corporate political philanthropy	Only domains
**Freudenberg**	2005	Public Health Advocacy to Change Corporate Practices: Implications for Health Education. Practice and Research	Essay	Literature	Industry that promote disease	Not specified	Corporations and public health generally	Advocacy Policy	Only domains
**Hancock**	2018	Applying Corporate Political Activity (CPA) analysis to Australian gambling industry submissions against regulation of television sports betting advertising.	Document analysis (qualitative)	Industry submissions to parliamentary committee inquiry	Gambling	Savells CPA gambling perspective	Specific regulation	CPA	Only recorded new findings
**Hastings**	2009	Tobacco corporate social responsibility and fairy godmothers: the Framework Convention on Tobacco Control slays a modern myth.	Short piece	FCTC	Tobacco	CSR	Tobacco CSR	CSR	Yes
**Hunt**	2020	How food companies use social media to influence policy debates: a framework of Australian ultra-processed food industry Twitter data	Review	Twitter data	Food, UP-food	Not specified	Twitter policy debates Australia	Influence debate, social media	No

**Jamieson**	2020	Oral health inequalities and the corporate determinants of Health: A commentary	Short piece	Literature	Corporations	Lukes three faces of power and Kickbusch	CDOH oral health	Corporate determinants of health CDOH	Yes
**Jones**	2016	Smokescreens and Beer Goggles: How Alcohol Industry CSM Protects the Industry	Case studies	CSM campaigns and literature	Tobacco, alcohol	Not specified	Alcohol CSR	Corporate social marketing, cause related marketing	No
**Kickbusch**	2016	The commercial determinants of health.	Comment (short piece)	WHO discussion paper	Private sector that provide products detrimental to health	Commercial determinants of health	Global health	Industrial epidemic, profit-driven, corporate practices	Yes
**Knai**	2018	Systems Thinking as a Framework for Analysing Commercial Determinants of Health.	Essay	Literature	Unhealthy commodity industries	Systems thinking framework (Donella Meadows)	UCIs and public health policy	UCI strategies, cohesive systems approach	No
**Knai**	2021	The case for developing a cohesive systems approach to research across unhealthy commodity industries.	Review	SRs, public information, company websites	Unhealthy commodity industries	CPA taxonomies	Commercial actors in policymaking	Corporate political strategies	No
**Leon**	2019	Legitimized fraud and the state-corporate criminology of food - a Spectrum-based theory	Theory development	Critical literature review	Food	Food fraud spectrum-based theory	Big food neoliberal governance	Fraud, criminology of food, neoliberal, hegemony	Yes
**Luo**	2017	Measuring Corporate Social Responsibility in Gambling Industry: Multi-Items Stakeholder Based Scales	Tool development	Literature and qualitative data	Gambling	CSR	Macau	CSR, responsible gambling	Yes
**Madureira Lima**	2018	Corporate practices and health: a framework and mechanisms.	Essay	Literature	TNCs	Luke’s three faces of power – dimensions, vehicles, practices and outcomes	Population health, policy	Macrosocial, corporate tactics	Yes
**Mariath**	2021	Sugary drinks taxation: Industry’s lobbying strategies, practices, and arguments in the Brazilian legislature	Content analysis (qualitative)	Two public hearings in Brazilian legislature	Food	INFORMAS (Mialon)	Brazil	CPA, lobbying	Only recorded new findings

**Matthes**	2021	Developing more detailed taxonomies of tobacco industry political activity in low-income and middle-income countries: qualitative evidence from eight countries.	Interviews (qualitative)	Interview transcripts	Tobacco	Policy dystopia model (Ulucunlar)	LMICs	LMICS, policy dystopia, political activities	Only recorded new findings
**McCambridge**	2018	Alcohol industry involvement in policymaking: a systematic review.	Review	Journal reports	alcohol	Savell conceptual model	Alcohol industry actors in policymaking	Corporate framing	Only recorded new findings
**McKee**	2018	Revisiting the corporate and commercial determinants of health.	Comment (short piece)	Concepts of CDOH literature	Corporations that determine health	The four ways corporations influence health	Global public health CDOH	CDOH, corporate power	No
**Mialon**	2020	An overview of the commercial determinants of health.	Review	Literature	Unhealthy commodities	Kickbusch	CDOH generally	CDOH, CPA	Yes
**Mialon**	2018	The policy dystopia model adapted to the food industry: the example of the Nutri-Score saga in France	Essay	Literature and observations	Food	Mialon and Ulucanlar Policy dystopia model	Specific policy Nutri-Score Saga, France	CPA	No
**Mialon**	2015	A proposed approach to systematically identify and monitor the corporate political activity of the food industry with respect to public health using publicly available information.	Review	Literature	food	CPA of the food industry	Public health policy	CPA	No
**Miller**	2010	Corporate strategy, corporate capture: Food and alcohol industry lobbying and public health.	Essay	Literature	Food and alcohol	Flex network and partnership governance	Public relations and lobbying	Corporate power, public relations	No
**Milsom**	2021	Corporate power and the international trade regime preventing progressive policy action on non-communicable diseases: a realist review	Review	Literature	Corporate power	Fusch and Lederer, Luke three dimensions, Madureira Lima and Galea	Transnational health-harmful corporations and power	Trade agreements, liberalization, political economy, power	Yes
**Moodie**	2013	Profits and pandemics: prevention of harmful effects of tobacco, alcohol, and ultra-processed food and drink industries.	Essay	Document analysis	Unhealthy commodities	Strategies by industry to undermine effective public health policies and programmes	Transnational corporations, international policy	Hard power, soft power	No

**Petticrew**	2020	Dark Nudges and Sludge in Big Alcohol: Behavioural Economics, Cognitive Biases, and Alcohol Industry Corporate Social Responsibility.	Review	Websites and materials of CSR organisations	Big alcohol	Existing Nudge conceptual frameworks: Hollands TIPPME and Thaler and Sunstein's ten types	Alcohol industry CSR and communications	Nudges, CSR	Yes
**Rochford**	2019	Reframing the impact of business on health: the interface of corporate, commercial, political and social determinants of health.	Short piece	Previous frameworks	Health impacting businesses	Conceptualisation of interactions between business and health	CDOH generally	CorDOH and ComDOH	Yes
**Savell**	2014	How does the tobacco industry attempt to influence marketing regulations? A systematic review.	Review	Literature	Tobacco	Hillman and Hitt 1999	Tobacco generally	Political activity, tactics, frames	No
**Scott**	2017	Food and beverage product reformulation as a corporate political strategy.	Content analysis (qualitative)	Literature, consultation analysis	Food and beverage	Taxonomy of food and beverage industry corporate political strategies	Nutritional reformulation as corporate political strategy	Political strategy, reformulation, framing, narratives	Yes Only recorded new findings
**Stillman**	2008	Mapping tobacco industry strategies in Southeast Asia for action planning and surveillance.	Concept mapping	Qualitative analysis of tobacco industry representatives	Tobacco	Concept mapping	Southeast Asia	Economic, politic, public relations, deception	Yes Only recorded new findings
**Thomas**	2016	Gambling advocacy: lessons from tobacco, alcohol and junk food	Interviews (qualitative)	Interviews	Gambling	Brownell and Warner 2009 strategies by big tobacco and food	Australia and New Zealand	Tactics, advocacy	Yes
**Trochim**	2003	Development of a model of the tobacco industry's interference with tobacco control programmes.	Concept mapping	34 expert consultations	Tobacco	Not specified	USA	Tactics	Yes
**Ulucanlar**	2016	The Policy Dystopia Model: An Interpretive Analysis of Tobacco Industry Political Activity.	Interpretive analysis (qualitative)	Literature	Tobacco	Taxonomy of discursive strategies and arguments used to construct policy dystopia	Tobacco generally	CPA	Yes
**Walls**	2020	Advancing alcohol research in low-income and middle-income countries: a global alcohol environment framework	Review	Literature	Alcohol	Previous CDOH conceptualisations	South Africa, LMICs 'local alcohol environment'	Environment, political, economic,	Yes Only recorded

								regulatory contexts	new findings
**Wood**	2021	Conceptualising the Commercial Determinants of Health Using a Power Lens: A Review and Synthesis of Existing Frameworks	Review	Framework literature	Health-harming industries	Integrated corporate power and health framework (Foucault: origins, nature and manifestations)	CDOH, corporate power generally	Corporate power, influence, CDOH, corporate strategy	Yes

Abbreviations: BAT: British American Tobacco, CDOH: Commercial Determinants Of Health, CHIA: Corporate Health Impact Assessment, COI: Conflict Of Interest, COR/COM: Corporate/Commercial, CPA: Corporate Political Activity, CSM/R: Corporate Social Marketing/Responsibility, FCTC: Framework Convention on Tobacco Control, LMIC: Low-Middle Income Country, SR: Systematic Review, TNC: Transnational Corporation, UCI: Unhealthy Commodity Industry, UP: Ultra-Processed, USA: United States of America, WHO: World Health Organisation.

Supplementary Table 7. List of example interactions between HCIs and English LAs. Retrieved during December 2021-February 2022.

Detail of interaction	Reference
The DH public-private partnership with industry to develop programmes intended to tackle the health problems arising from the products those same industries manufacture or distribute.	Department of Health (2011) Public health responsibility deal. Gov.UK. https://www.gov.uk/government/news/public-health-responsibility-deal
The Drinkaware Trust, a government-industry partnership on public education campaigns was established by the Portman group	Portman Group (2021) Portman Group calls on WHO for renewed focus on harmful drinking and to recognise the industry as a constructive partner. Portmangroup.org. https://www.portmangroup.org.uk/portman-group-calls-on-who-for-renewed-focus-on-harmful-drinking-and-to-recognise-the-industry-as-a-constructive-partner/
Previously 'Change4Life', the 'Better health, healthier families' initiative is the DOH anti-obesity campaign, partners include unhealthy industries	Better Health, healthier families (2021) Easy ways to eat well and move more. NHS.UK. https://www.nhs.uk/healthier-families/.
The 'Childhood Obesity Trailblazer Programme' (COTP) Sugar Smart campaign is a joined force between Lewisham Council and JCDecaux (partnered with, e.g. Coca-cola and McDonalds)	Lewisham Council (2020) Lewisham Council has joined forces with outdoor advertising companies to tackle childhood obesity. Lewisham.gov.uk. https://lewisham.gov.uk/articles/news/lewisham-council-has-joined-forces-with-outdoor-advertising-companies-to-tackle-childhood-obesity
The Mayor of London and Transport for London (TFL) advertisement policy banning junk food advertising on the entire TFL network	Transport for London (2019) TFL.gov.uk. https://content.tfl.gov.uk/policy-guidance-food-and-drink-advertising.pdf
Primary Authority Partnership between Birmingham City Council and Cadbury Mondelez International, which meant a waiver and cut of the cost of regulatory expenses for packaging changes (£250,000) to reduce unnecessary burden on business	Better Regulation Delivery Office (2014) Primary Authority: A guide for Officials. Department for Business Innovation & Skills. Gov.uk https://assets.publishing.service.gov.uk/government/uploads /system/uploads/attachment_data/file/583872/14-1058-pa-guide-for-officials.pdf
Westminster City Council inferring their intention to seek commercial opportunities to make up for reduction in central government grants	Mair, S. (2016) Westminster City Council Annual Accounts 16/17. Westminster.go.uk ttps://www.westminster.gov.uk/sites/default/files/annual_accounts_2016-17.pdf.
Coca-Cola Christmas truck permission from LA's to park, host and promote their products in the locality	Sustain (2018) UK loses the taste for the Coke truck . Sustainweb.org. https://www.sustainweb.org/news/nov18_coke_truck/
Westminster, alongside City of London were in the LAs with the most betting shops per capita in England. Gambling outlets are in most deprived communities, near gambling treatment centres and schools	Evans, J. and Cross, K. (2021). The Geography of Gambling Premises in Britain. Abrdn.com https://www.abrdn.com/docs?editionId=c8d6f9b5-1c8b-4b97-9bb4-c3099938f737
Scheme as part of the London Healthier Catering Commitments Project encourages food outlets in London to voluntarily apply for an award to commit to offering healthier options	Haringey London (2022). Healthier Catering Commitment. Haringey.gov.uk https://www.haringey.gov.uk/social-care-and-health/health/public-health/healthy-haringey/healthier-catering-commitment & https://healthiercateringcommitment.co.uk/
Coca-Cola investments in the APPG (all-party parliamentary group) partnership with LAs, on a fit and healthy childhood, promotes information about childhood health, including obesity, to inform policy	Coca-Cola GB (2020) Our investments in health and wellbeing research and partnerships – organisations. Coca-cola.co.uk https://www.coca-cola.co.uk/our-business/company-statements/our-investments-in-health-and-wellbeing-research-and-partnerships
Croydon Council purchase of a retail park home to Nando's and McDonalds, to create funding for service and generate income	Krause, R. (2019) Council purchases Nando's and McDonald's stores as part of bid to 'protect frontline services'. Yourlocalguardian. https://www.yourlocalguardian.co.uk/news/17831852.council-purchases-nandos-mcdonalds-stores-part-bid-protect-frontline-services/

Hampshire County Council pension funds in tobacco sector	Shaw, A. (2017) Hampshire County Council slammed after £80 million pumped into British American Tobacco. Daily Echo. https://www.dailyecho.co.uk/news/15323523.hampshire-county-council-slammed-after-80-million-pumped-into-british-american-tobacco/
Kingston Upon Thames pension fund in industries such as Coca-Cola	Pension Fund Panel (2012) Pension Fund Annual Report 2011/12. Kingston.gov.uk Enclosure 2 draft Pension Fund Annual report (kingston.gov.uk)
Stratford Council awarding money to Starbucks to improve the appearance of its outlet on the town's historic area	Lugg, B. (2017) Questions raised over council grant to Starbucks. Stratford Herald. https://www.stratford-herald.com/news/questions-raised-over-council-grant-to-starbucks-9136669/
'Project open' personalised business support from Coca-Cola to Lewisham small businesses ('post lockdown local business support')	Fosker, A. (2021) Two Spoons Receives Tailored Support from Coca-Cola to Aid Business Recovery. LewishamLocal. https://www.lewishamlocal.com/two-spoons-receives-tailored-support-from-coca-cola-to-aid-business-recovery/<br>Coca-Cola Project Open: https://www.coca-cola.co.uk/community/project-open
The Community Alcohol Partnership charity funded by alcohol sellers, linking them to local communities	CAP (2020) Community Alcohol partnerships Annual Report 2020. https://www.communityalcoholpartnerships.co.uk/images/documents/CAP-Annual-Report-2020-9.pdf
'Keep Britain Tidy' partners include Nestle, KFC, McDonalds, Walkers, Pepsi, keep regular communications with members, including LAs – e.g. Wakefield Council (quoted)	Keep Britain Tidy (2022). Become a member of the keep Britain tidy network. Keepbritaintidy.org. https://www.keepbritaintidy.org/local-authorities/become-a-member
The 'Neighbourly Community Fund' and 'neighbourly foundation' links industries including Coca-Cola and Heineken with the local community	Neighbourly (2022) Where businesses help local good causes. Neighbourly.com https://www.neighbourly.com/
Sponsorship agreement between Coca-Cola and Westminster City Council for exclusive rights, licenses and opportunities for sponsorship, advertising, and promotional rights in relation to the recycling bins in public spaces	Westminster City Council (2009) SA Coca-Cola Services NV and Westminster City Council Sponsorship Agreement. whatdotheyknow.com ttps://www.whatdotheyknow.com/request/120266/response/298881/ attach/3/Recycling%20Bin%20Sponsorship%20Agreement.pdf?cookie_passthrough=1
Football, premier league, and gambling	BBC News (2018) Does football have a gambling problem? BBC https://www.bbc.co.uk/bbcthree/article/b542778d-871f-4716-abfb-0afb91fa7770
Cadbury Premier League supporting the local community in which the team belongs – arsenal (Islington local support), Chelsea, Tottenham	Sport Industry Group (2020) Arsenal partner with Cadbury to support local community. Sportindustry.biz https://www.sportindustry.biz/news/arsenal-partner-cadbury-support-local-community
Coca-Cola actions on society, including the Community Pub Fund, Youth's Reach Up Programme, FareShare food redistribution	The Coca-Cola company (2021) Actions on society (our communities). Coca-Cola. https://www.cocacolaep.com/assets/Sustainability/Documents/1ec8a40410/2020-Action-on-Society-Our-Communities-factsheet.pdf
Tottenham council promoting the use of a joint project with a fried chicken shop to develop and test a new healthier menu	Public Health England (2018) Healthy High Streets Challenge and Tasters Fried Chicken Shop. Gov.uk https://www.gov.uk/government/case-studies/healthy-high-streets-challenge-and-tasters-fried-chicken-shop
The Social Issues Research Centre (SIRC) carries out research on lifestyle issues such as diet, funded by (e.g.) Coca-Cola, Cadbury, Schweppes	Social Issues Research Centre (2022) SIRC http://www.sirc.org/about/about.html

Coca-Cola Pub Fund to reward a small business or fund a community project as well as a donation to a local charity. The pub award fund was used to refurbish the Victoria Newton in St. Helens Borough	Mulligan, S. (2021) Coca Cola fund outdoor refurbishment at The Victoria, Newton. St Helens Star. https://www.sthelensstar.co.uk/news/19246447.coca-cola-fund-outdoor-refurbishment-victoria-newton/
Coca-Cola GB ParkLives partnership with LAs and charity partner StreetGames, investing in pitches and coaches across UK cities	Coca-Cola GB (2022) Ball games allowed. Coca-Cola Great Britain. https://www.coca-cola.co.uk/community/streetgames/ball-games-allowed
Camborne Town Council promoting Streetgames	Camborne Town Council (2022) ParkPlay Camborne Free Activity Mornings. Parkplay. Events Camborne Town Council ps://www.camborne-tc.gov.uk/events/parkplay-camborne-free-activity-mornings
Birmingham City Council promoting ParkLives and direct relation to Coca-Cola	Birmingham City Council (2017) Fun, free outdoor activities on offer with Parklives return. Birmgingham.gov https://www.birmingham.gov.uk/news/article/66/fun_ free_outdoor_activities_on_offer_with_parklives_return
Cadbury campaign to drive customers to purchase locally, playing to their origins as a small business, and to benefit their partnerships with high street brands	McCarthy, J. (2021) Cadbury urges support for high street chocolate stores with rework of 'Mum's Birthday' ad. The Drum News. https://www.thedrum.com/news/2021/04/28/cadbury-urges-support-high-street-chocolate-stores-with-rework-mum-s-birthday-ad
Suffolk council endorsing the Change4Lfie campaign	Suffolk County Council (2016) Parents encouraged to get Sugar Smart. Suffok.gov https://www.suffolk.gov.uk/council-and-democracy/council-news/show/parents-in-suffolk-encouraged-to-get-sugar-smart
Coca-Cola consultancy, educational grants, donations and membership of British Nutrition Foundation	Coca-Cola GB (2020) Our investments in health and wellbeing research and partnerships – organisations. Coca-cola.co.uk tps://www.coca-cola.co.uk/our-business/company-statements/our-investments-in-health-and-wellbeing-research-and-partnerships
Coca-Cola investment and partnership in British Dietic Association regarding evidence review on Stevia	Coca-Cola GB (2020) Our investments in health and wellbeing research and partnerships – organisations. Coca-cola.co.uk tps://www.coca-cola.co.uk/our-business/company-statements/our-investments-in-health-and-wellbeing-research-and-partnerships
Coca-Cola partnership with Nova International Conference on Healthy eating and Physical activity	Coca-Cola GB (2020) Our investments in health and wellbeing research and partnerships – organisations. Coca-cola.co.uk tps://www.coca-cola.co.uk/our-business/company-statements/our-investments-in-health-and-wellbeing-research-and-partnerships
Coca-Cola industry membership in the Diet and Health Research Industry Club (DRINC), which funds research about diet and health	UKRI (2022) The Diet and Health Research Industry Club (DRINC). https://www.ukri.org/about-us/bbsrc/who-we-are/sharing-challenges/drinc/
BET365 support young people's gambling harm prevention programme, including education, treatment and community projects	Patel, J. (2021) BGC praises gambling industry's "huge contribution" across corporate social responsibility initiatives. Gambling Spotlight. https://www.gamblingspotlight.com/bgc-praises-gambling-industrys-huge-contribution-across-corporate-social-responsibility-initiatives/
The Cadbury Foundation, mostly through grant making, operating in UK LAs, for example, Birmingham and City of London	Charity Commission for England and Wales (2022) The Cadbury Foundation. Charity Commission https://register-of-charities.charitycommission.gov.uk/charity-search/-/charity-details/1050482/what-who-how-where
Cadbury Mondelez foundation awards funding to community studio in Oswestry Town	Mondelez International (2021) Local community studio receives £5,000 from The Cadbury Foundation. My news desk.com https://www.mynewsdesk.com/uk/mondelez-uk/pressreleases/local-community-studio-receives-5000-pounds-from-the-cadbury-foundation-3107455

Pepsi acquired Quaker, which is partnered with the charity 'Magic Breakfast', bringing breakfast to children across the UK	Molyneux, C. (2021) Quaker and Pro Green join forces with magic breakfast. Pepsico.co.uk https://www.pepsico.co.uk/news/stories/quaker-and-pro-green-join-forces
Mars strategic partnership with 'KIND' brand as part of health drive – healthy and philanthropic social responsibility initiatives	KIND (2022) KIND and Mars Announce Next Step in Partnership to Build a Kinder World and Bring Healthy Snacks to People Worldwide. Kindsnacks. https://www.kindsnacks.com/kind-mars-partnership.html
Gov.uk promoting the magic breakfast activities	Department for Education & Ford, V. (2020). Thousands of children offered nutritious breakfasts during summer. Gov.uk https://www.gov.uk/government/news/thousands-of-children-offered-nutritious-breakfasts-during-summer
Coca-Cola London Eye promoted on Lambeth Council website	Lambeth Council (2022) The Coca Cola London Eye. Lambeth.gov. https://beta.lambeth.gov.uk/choose-love/venues/licensed-venues/coca-cola-london-eye
McDonalds 'good times' summer activities promoted on Lambeth Council website	Lambeth Council (2016) McDonald's, Good Times Brand Summertime Activation. Lambeth.gov. https://www.lambeth.gov.uk/events/mcdonalds-good-times-brand-summertime-activation
Lewisham collaborative programme to ban HFSS food advertising from council owned advertising spaces	Witton, B. (2019) Junk food advertising ban set to help Lewisham fight obesity. News Shopper. https://www.newsshopper.co.uk/news/17393374.junk-food-advertising-ban-set-help-lewisham-fight-obesity/
The Betting and Gaming Council issues warning about the dangers posed by the unregulated and illegal online black market in gambling. Members of the BGC include 90% of all the UK's licensed gambling businesses (including online gaming)	The BGC (2022) The Betting and Gaming Council issues warning about the dangers posed by the unregulated and illegal online black market in gambling. The Betting and Gaming Council. https://bettingandgamingcouncil.com/campaigns/the-betting-and-gaming-council-issues-warning-about-the-dangers-posed-by-the-unregulated-and-illegal-online-black-market-in-gambling
Westminster McDonalds Franchisee, Capital Arches Group Ltd, owned by Claude Abi-Gerges, promotes connections with community and LA	Westminster (2020) Capital Arches Group – Your Local McDonald's Franchisee. Westminster.gov. https://committees.westminster.gov.uk/documents/s34780/3.1j%20-%20CAG%20Bio.pdf
McDonald's litigation case against Newport Carmarthenshire Council	Youle, R. (2020) Council facing costs bill after rejecting drive-thru McDonald's and Costa coffee development. Wales online. https://www.walesonline.co.uk/news/wales-news/planning-costs-carmarthenshire-council-news-18401531
Bristol Council refused to partner with Clean up Britain campaign, which receives funding from Phillip Morris Tobacco	Bloch, B. (2022) Drones and AI being used to clean up Bristol. Bristol Post. https://www.bristolpost.co.uk/news/bristol-news/drones-ai-being-used-clean-6526063?utm_source=twitter.com&utm_medium=social&utm_campaign=sharebar
Tobacco acting as the solution, with illicit trade campaigns which links them with local police, and reduce necessity for funding to go into enforcing illicit trade	Phillip Morris International (2022) Collaboration remains the key to success in the fight against illicit trade. PMI.com https://www.pmi.com/illicit-trade-prevention/collaboration-remains-the-key-to-success-in-the-fight-against-illicit-trade
LA investment portfolios and commercial borrowing (e.g. from banks, building societies) which also have harmful commodity industries as clients	National Audit Office (2020) Local authority investment in commercial property. NAO.org https://www.nao.org.uk/report/local-authority-investment-in-commercial-property/
Asda supermarket (which sells harmful products e.g. alcohol) owned by Mole Valley District Council Dorking. The council receives annual rental income	West, K. (2019) The supermarket in Wales owned by a small council…in Surrey. The guardian. https://www.theguardian.com/business/2019/jan/26/small-supermarket-wales-owned-surrey-casino-property

Spelthorne Borough Council bought BP on Sunbury-on Thames campus	West, K. (2019) The supermarket in Wales owned by a small council…in Surrey. The guardian. https://www.theguardian.com/business/2019/jan/26/small-supermarket-wales-owned-surrey-casino-property
Court overturns Local Government Pension Scheme investment ban from companies which profit from harmful products	UNISON (2020) UNISON welcomes Supreme Court decision on ethical LGPS investment. Unison.org. https://www.unison.org.uk/news/article/2020/04/lgps-court/
LA services outsourced to third party providers with harmful industry links / influenced by industry (e.g. Capital Public Service)	Capita (2022) Improving lives with efficient local government services. Capita.com https://www.capita.com/industries/government/local-government
Burger King franchise long-term contract with Cambridge Addenbrooke hospital	NHS Cambridge University Hospitals (2022) Shops, Cafes and restaurants. Cuh.NHS.uk https://www.cuh.nhs.uk/visiting-our-hospitals/shops-cafes-restaurants/
Diabetes UK partnership with Britvic (brands include Mountain Dew, Pepsi, tango, Whites) to support children's educational programmes	Diabetes UK (2018) We've joined forces with Britvic in a three-year partnership. Diabetes.org.uk https://www.diabetes.org.uk/about-us/news/britvic-three-year-partnership

Abbreviations: LA: Local Authority, PR: Public Relations, BC: Borough Council, CC: County/City Council, NGO: Non-Government Organisation, DOH: Department Of Health, PHE: Public Health England, HFSS: foods High in Fat, Sugar and Salt .
